# Relationship of body mass index with frailty and all-cause mortality among middle-aged and older adults

**DOI:** 10.1186/s12916-022-02596-7

**Published:** 2022-10-24

**Authors:** Kulapong Jayanama, Olga Theou, Judith Godin, Andrea Mayo, Leah Cahill, Kenneth Rockwood

**Affiliations:** 1 Chakri Naruebodindra Medical Institute, Faculty of Medicine Ramathibodi Hospital, Mahidol University, Samut Prakan, Thailand; 2 Division of Geriatric Medicine, Dalhousie University & Nova Scotia Health, Halifax, Nova Scotia Canada; 3 School of Physiotherapy, Dalhousie University, Halifax, Nova Scotia Canada; 4 Department of Medicine, Dalhousie University, Halifax, Nova Scotia Canada; 5 Department of Nutrition, Harvard T.H. Chan School of Public Health, Boston, MA USA

**Keywords:** Body mass index, Obese, Body composition, Percent body fat, Frailty, Mortality

## Abstract

**Background:**

Parallel to growth of aging and obese populations, the prevalence of metabolic diseases is rising. How body mass index (BMI) relates to frailty and mortality across frailty levels is controversial. We examined the associations of high BMI with frailty and mortality and explored the effects of percent body fat on these associations.

**Methods:**

We included 29,937 participants aged ≥50 years from the 2001–2006 National Health and Nutrition Examination Survey (NHANES) cohorts (*N*=6062; 53.7% females) and from wave 1 (2004) of Survey of Health, Ageing and Retirement in Europe (SHARE) (*N*=23,875; 54% females)*.* BMI levels were categorized as: normal: 18.5–24.9 kg/m^2^, overweight: 25.0–29.9, obese grade 1: 30.0–34.9, and obese grade 2 or 3: >35.0. A frailty index (FI) was constructed excluding nutrition-related items: 36 items for NHANES and 57 items for SHARE. We categorized the FI using 0.1-point increments: FI ≤ 0.1 (non-frail), 0.1 < FI ≤ 0.2 (very mildly frail), 0.2 < FI ≤ 0.3 (mildly frail), and FI > 0.3 (moderately/severely frail). Percent body fat was measured using DXA for NHANES participants. All-cause mortality data were obtained until 2015 for NHANES and 2017 for SHARE to estimate 10-year mortality risk. All analyses were adjusted for age, sex, educational, marital, employment, and smoking statuses.

**Results:**

Mean age of participants was 63.3±10.2 years for NHANES and 65.0±10.0 years for SHARE. In both cohorts, BMI levels ≥25 kg/m^2^ were associated with higher frailty, compared to normal BMI. In SHARE, having a BMI level greater than 35 kg/m^2^ increased mortality risk in participants with FI≤0.1 (HR 1.31, 95%CI 1.02–1.69). Overweight participants with FI scores >0.3 were at lower risk for mortality compared to normal BMI [NHANES (0.79, 0.64–0.96); SHARE (0.71, 0.63–0.80)]. Higher percent body fat was associated with higher frailty. Percent body fat significantly mediated the relationship between BMI levels and frailty but did not mediate the relationship between BMI levels and mortality risk.

**Conclusions:**

Being overweight or obese is associated with higher frailty levels. In this study, we found that being overweight is a protective factor of mortality in moderately/severely frail people and obesity grade 1 may be protective for mortality for people with at least a mild level of frailty. In contrast, obesity grades 2 and 3 may be associated with higher mortality risk in non-frail people. The relationship between BMI and frailty is partially explained by body fat.

**Supplementary Information:**

The online version contains supplementary material available at 10.1186/s12916-022-02596-7.

## Background

Populations are growing older. With this, changes in patterns of health can be expected, but the impacts of these changes are not yet clear. With aging, it is likely that there will be increases in many important types of ill health, such as impaired cognition, or disability. With dementia, for example, net increases in the number of cases may occur even if incidence or age-specific prevalence rates fall [1, 2]. Similar trends are seen in relation to disability [3] as well as frailty, which is expected to increase even as lethality might diminish [4, 5].

One important change in health is expected to be a growing proportion of people with obesity [6, 7]. In the USA, middle-aged and older individuals have a higher prevalence of obesity compared to young adults [8]. To determine body fatness or obesity, body mass index (BMI) or Quetelet index (calculated by body weight (kg) divided by height squared (m^2^)) is generally used [9]. Even so, BMI may not discriminate between lean and fat mass [10]. In addition, the association between BMI and body composition differs among races [11], age groups [12], and people with underlying diseases such as chronic kidney disease and cancer [13, 14]. In older individuals, high BMI is related to increased comorbid diseases [15] and poor physical function, social health, and quality of life [16, 17]. On the contrary, higher BMI is associated with higher bone mineral density and lower incidence of hip fracture due to weight-bearing and hormonal osteoprotective effects [18]. Therefore, the impact of obesity on health outcomes in older people is complex; this has been described as the obesity paradox [19]. Weight reduction improves obesity-related metabolic abnormalities, physical function, and quality of life [7], whereas it is related to less bone and fat-free mass and a higher risk of osteoporotic fracture and sarcopenia in older, obese, and inactive people [20].

Frailty in relation to obesity is also a complex topic. Two approaches to frailty are recognized; one views it as a shrinking syndrome (of weight loss, reduced activities, slower gait speed, worse grip strength, and exhaustion) [21]. The other views frailty as the accumulation of deficits [22] and is a more holistic approach. Weight gain has been described in some frail people resulting from shrinkage (on the grounds of muscle loss), consistent with sarcopenic obesity [23].

Few studies [24–26] have examined the relationship between BMI and frailty, and their findings are contradictory; therefore, the relationship between the obesity paradox and frailty remains unknown. Furthermore, the association between BMI and health outcomes differs by frailty status [26]. Our objectives were (1) to examine the association of high BMI with frailty, (2) to evaluate the impact of high BMI on mortality risk when stratified by the degree of frailty, and (3) to explore the effect of percent body fat measured with dual-energy X-ray absorptiometry (DXA) on these relationships.

## Methods

### Study population and design

This observational study analyzed the data from two cohorts: the National Health and Nutrition Examination Survey (NHANES) and the Survey of Health, Ageing and Retirement in Europe (SHARE)*.* The analyzed data were deidentified and publicly available.

#### National Health and Nutrition Examination Survey (NHANES)

NHANES is a series of publicly available, cross-sectional surveys focusing on the health and nutrition of non-institutionalized US residents [27, 28]. Here, we included participants aged 50 years or older from the 2001–2002, 2003–2004, and 2005–2006 cohorts who had a BMI of 18.5 kg/m^2^ or more. We excluded participants with BMI <18.5 kg/m^2^ due to low prevalence (N =78 (1.2%)). Participants with missing Frailty index (FI) scores (*N* =246) and mortality (*N* =9) data were also excluded, leaving a total of 6062 participants. In NHANES, weight and height, measured by well-trained study personnel, were used to calculate BMI levels. To explore the third objective of our study, percent body fat was measured in 5310 participants using DXA. Whole-body DXA scans were taken with a Hologic QDR-4500A fan-beam densitometer (Hologic, Inc., Bedford, MA) while participants were in supine and neutral position. Each participant provided written informed consent. The NHANES protocol was approved by the institutional review board of the Centers for Disease Control and Prevention (CDC).

#### Survey of Health, Ageing and Retirement in Europe (SHARE)

SHARE is a longitudinal, multinational, representative health survey of community-dwelling Europeans aged 50 years or older. Here, we included participants aged 50 years or older from wave 1 with BMI 18.5 kg/m^2^ or more (release version 7.0.0, April 3rd, 2019)*.* We excluded participants with BMI <18.5 kg/m^2^ due to low prevalence (*N* =293 (1.2%)). Wave 1 of SHARE was conducted in 2004-2005 and the participating countries were Austria, Belgium, Denmark, France, Germany, Greece, Israel, Italy, Netherlands, Spain, Sweden, and Switzerland [29, 30]. Participants lost to follow-up (*N* =5093) and with missing FI scores (*N* =165) and mortality (*N* =235) data were excluded, leaving a total of 23,875 participants. To examine the relationship of BMI with frailty longitudinally, we also analyzed data from 11,136 participants who had complete FI data in wave 6 (2014–2015). Self-reported weight and height data were used to calculate BMI levels. The SHARE project was approved by the Ethics Committee of the University of Mannheim [31]. The SHARE data collection procedures are subject to continuous ethics review by international research ethics principles such as the professional and ethical guidelines for the conduct of socio-economic research and the Declaration of Helsinki.

### Outcome: Frailty

In both datasets, frailty was evaluated using an FI. This outcome was examined cross-sectionally in NHANES and both cross-sectionally and longitudinally at 10 years follow-up in SHARE. In NHANES, we used a modified FI from previous NHANES studies using 36 items which were available in all included cohorts [32–34]; 10 items of the original 46 items related to intake or nutritional status were excluded (Additional file 1: Table S1). In SHARE, we used a 57-item FI which was modified from a previously validated 70-item FI [35]; two nutrition intake-related items and 11 items missing from the SHARE wave 6 were excluded (Additional file 1: Table S2). For both datasets, the FI score was calculated by the number of deficits present divided by the total deficits considered. FI scores ranged between 0 and 1; a higher score indicates higher frailty. For stratification purposes, we categorized participants into 4 groups per 0.1-point FI score increments: FI ≤ 0.1 (non-frail), 0.1 < FI ≤ 0.2 (very mildly frail), 0.2 < FI ≤ 0.3 (mildly frail), and FI > 0.3 (moderately/severely frail) [32, 36].

### Outcome: mortality

For both datasets, 10-year all-cause mortality risk was examined. In NHANES, mortality status was identified from the death certificate records from the National Death Index in December 31, 2015. Survival time was counted from the date of the clinical examination to the death event up to 10 years. In SHARE, mortality status up to 10 years was recorded from all waves up to wave 7 (2017) and death status and date of death were reported by a proxy respondent (a family member, household member, or neighbor).

### Statistical analysis

For both datasets, participant characteristics stratified by BMI levels were presented as mean ±standard deviation (SD) for continuous variables or as frequency (%) for binary or categorical variables. All percentages and mean values were weighted using the sampling weights provided by each dataset. Participants were classified into 4 groups by BMI: 18.5–24.9 kg/m^2^ (normal weight), 25.0–29.9 (overweight), 30.0–34.9 (obese grade 1), and ≥35.0 (obese grade 2 and 3) [37, 38]. Regarding the first objective, the association between BMI and FI was analyzed using ordinary least squares (OLS) regression analysis and is presented in unstandardized beta-coefficients with corresponding 95% confidence intervals (CIs). For the second objective, the mortality risk of having a higher BMI was assessed using Cox regression models stratified by FI levels, and mortality risk is presented using hazard ratios (HR) and the associated 95% CIs. The interactions of age and sex with BMI levels in relation to frailty, and of age, sex, and frailty with BMI levels in relation to mortality risk were also tested in the OLS and Cox regression models. With regard to the last objective, the association between BMI and percent body fat was tested using Pearson’s correlation (r). The mediating effects of percent body fat on the relationship between BMI and frailty was examined using the single-mediation regression model by Andrew F. Hayes [39], while its effects on the relationship between BMI and mortality risk were examined using the product of coefficients approaches by MacKinnon [40]. All regression models were adjusted for available potential covariates, including age (continuous in year), sex (male and female), marital status (married, widowed, divorced or separated, or never married), employment status (working or non-working), smoking (NHANES: never, former, or current; SHARE: no or yes), and education levels (NHANES: less than high school, high school, some college/associated education, and college graduate or more). SHARE employed the International Standard Classification of Education (ISCED), which assigned categorical levels of education ranging from 0 (pre-primary education) to 6 (Second stage of tertiary education) [41]: none or Isced-97 code 1-2, Isced-97 code 3, Isced-97 code 4, and Isced-97 code 5-6.

Statistical significance was considered as a *p* <0.05 and all reported probability tests were two-tailed. The statistical analysis was conducted using IBM SPSS Statistics Version 25.0. Armonk, NY: IBM Corp.

## Results

In both NHANES and SHARE, just over half of the participants were female (53.7% and 54%, respectively). In NHANES, the weighted mean age and BMI were 63.3 ±10.2 years (35.4% older than 65 years) and 28.9 ±6.0 kg/m^2^ (34.7% being obese), respectively; the weighted median (IQR) of FI was 0.12 (0.07–0.22). In SHARE, the weighted mean age and BMI were 65.0 ±10.0 years (43.9% older than 65 years) and 26.7 kg/m^2^ (17.9% being obese), respectively; the weighted median (IQR) of FI at wave 1 and at wave 6 were 0.13 (0.07–0.22) and 0.16 (0.09–0.27), respectively. The 10-year mortality rate was 25.3% (93.5 cases per 1000 person-years) in NHANES and 16.1% (21.5 cases per 1000 person-years) in SHARE (data not shown in Tables and Figures). In both datasets, participants with higher BMI tended to be younger, female, less educated, divorced/separated or never married, and non-working, and tended to have lower income and higher FI scores (Table 1).

**Table 1:** Descriptive characteristics of participants by body mass index

Characteristics	Body mass index (kg/m^**2**^)							
	NHANES (***N*** =6062)				SHARE (***N*** =23,875)			
	18.5–24.9	25.0–29.9	30.0–34.9	≥35.0	18.5–24.9	25.0–29.9	30.0–34.9	≥35.0
*N* (%)	1656 (27.6)	2339 (37.7)	1264 (20.6)	803 (14.1)	9397 (39.4)	10,197 (42.7)	3371 (14.1)	910 (3.8)
Age (year), mean ±SD	64.3 ±11.2	64.8 ±10.3	62.7 ±9.6	60.9 ±8.4	65.1 ±10.7	65.0 ±9.7	65.1 ±9.5	63.2 ±9.0
Sex, female, *N* (%)	899 (62.0)	1002 (45.8)	621 (46.7)	514 (64.3)	5672 (61.6)	4774 (47.6)	1843 (56.0)	605 (67.1)
Education, *N* (%)								
Less than high school	527 (20.5)	774 (20.5)	427 (21.9)	272 (20.7)	4396 (47.5)	5495 (53.3)	2037 (60.8)	579 (63.8)
High school	400 (26.0)	542 (25.9)	323 (27.3)	193 (28.1)	2529 (28.9)	2656 (28.9)	848 (27.4)	205 (24.2)
Some college/associated education	369 (24.3)	564 (28.7)	284 (26.4)	226 (32.8)	289 (1.9)	228 (1.6)	81 (1.4)	22 (1.2)
College graduate or more	355 (29.1)	454 (24.8)	229 (24.2)	112 (18.3)	2183 (21.7)	1818 (16.2)	405 (10.4)	104 (10.8)
Annual household income (USD), *N* (%)								
0–19,999	449 (20.6)	534 (16.0)	330 (18.8)	203 (20.8)	2644 (31.3)	3229 (34.2)	1249 (40.8)	382 (45.9)
20,000–44,999	505 (30.3)	772 (32.0)	383 (31.7)	268 (33.2)	2909 (32.7)	3196 (33.5)	1037 (33.1)	295 (32.4)
45,000–74,999	287 (22.5)	436 (24.9)	240 (23.6)	152 (25.0)	2100 (20.1)	2151 (19.2)	639 (15.8)	133 (13.7)
≥75,000	283 (26.6))	421 (27.1)	214 (25.9)	121 (21.0)	1743 (15.9)	1621 (13.1)	446 (10.4)	101 (8.0)
Marital status, *N* (%)								
Married	963 (63.5)	1529 (69.9)	794 (67.9)	473 (64.4)	6587 (64.1)	7640 (69.9)	2423 (65.7)	610 (59.9)
Widowed	362 (16.0)	441 (14.6)	234 (15.3)	133 (11.9)	1483 (19.2)	1432 (17.1)	561 (21.1)	146 (21.2)
Divorced or separated	248 (15.9)	289 (12.6)	186 (13.3)	144 (17.3)	753 (8.7)	660 (6.9)	231 (7.2)	99 (11.3)
Never married	81 (4.5)	77 (2.7)	49 (3.2)	53 (6.4)	573 (8.0)	466 (6.2)	157 (6.0)	55 (7.6)
Full-time working, *N* (%)	534 (43.4)	860 (48.2)	455 (47.5)	300 (46.7)	2878 (29.1)	2938 (26.3)	817 (20.4)	213 (20.3)
Smoking status, *N* (%)								
Never	772 (47.0)	1033 (44.6)	583 (45.9)	393 (48.5)	4998 (56.2)	5258 (54.5)	1851 (59.8)	526 (60.3)
Former	531 (30.8)	986 (40.9)	491 (37.8)	314 (39.0)	4399 (43.8)	4939 (45.5)	1520 (40.2)	384 (39.7)
Current	350 (22.2)	317 (14.5)	190 (16.3)	94 (12.5)				
Frailty index score (baseline), median (IQR) and mean ±SD	0.11 (0.06–0.20) 0.14 ±0.12	0.12 (0.06–0.20) 0.15 ±0.11	0.13 (0.08–0.23) 0.17 ±0.12	0.18 (0.11–0.29) 0.21 ±0.13	0.11 (0.06–0.19) 0.16 ±0.13	0.12 (0.06–0.19) 0.17 ±0.13	0.16 (0.09–0.26) 0.21 ±0.14	0.20 (0.19–0.32) 0.24 ±0.15
Frailty index score (follow-up), median (IQR) and mean ±SD	-	-	-	-	0.13 (0.07–0.23) 0.18 ±0.15	0.13 (0.07–0.23) 0.21 ±0.14	0.20 (0.11–0.32) 0.25 ±0.16	0.26 (0.16–0.36) 0.29 ±0.15
10-year mortality rate	655 (28.8)	790 (24.1)	363 (22.8)	225 (25.3)	1630 (17.1)	1579 (14.4)	573 (17.5)	156 (17.5)

The percentages and mean values are weighted

*kg* kilogram, *m* meter, *USD* United States Dollar (1 USD = 0.89 Euro)

### Objective 1: association of high BMI with frailty

**Fig. 1:**
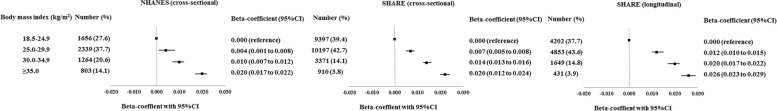
Relationship between body mass index and frailty. All regression models were adjusted for age, sex, educational level, marital status, employment status, and smoking. Cross-sectional analysis examined the body mass index and frailty index from the NHANES 2001–2006 cohorts and SHARE wave 1. Longitudinal analysis examined the body mass index from SHARE wave 1 and frailty index from SHARE wave 6

We did not find a significant interaction of age and sex with BMI levels in relation to frailty. After controlling for covariates, all BMI levels greater than 25 kg/m^2^ (25.0–29.9, 30.0–34.9, and ≥35.0 kg/m^2^) were statistically significantly associated with higher frailty cross-sectionally and longitudinally, compared to those with normal BMI (18.5–24.9 kg/m^2^); the higher BMI group had statistically significantly higher frailty levels in both datasets (*p* for trend <0.001) (Fig. 1).

### Objective 2: impact of high BMI on mortality risk when stratified by degree of frailty

**Fig. 2:**
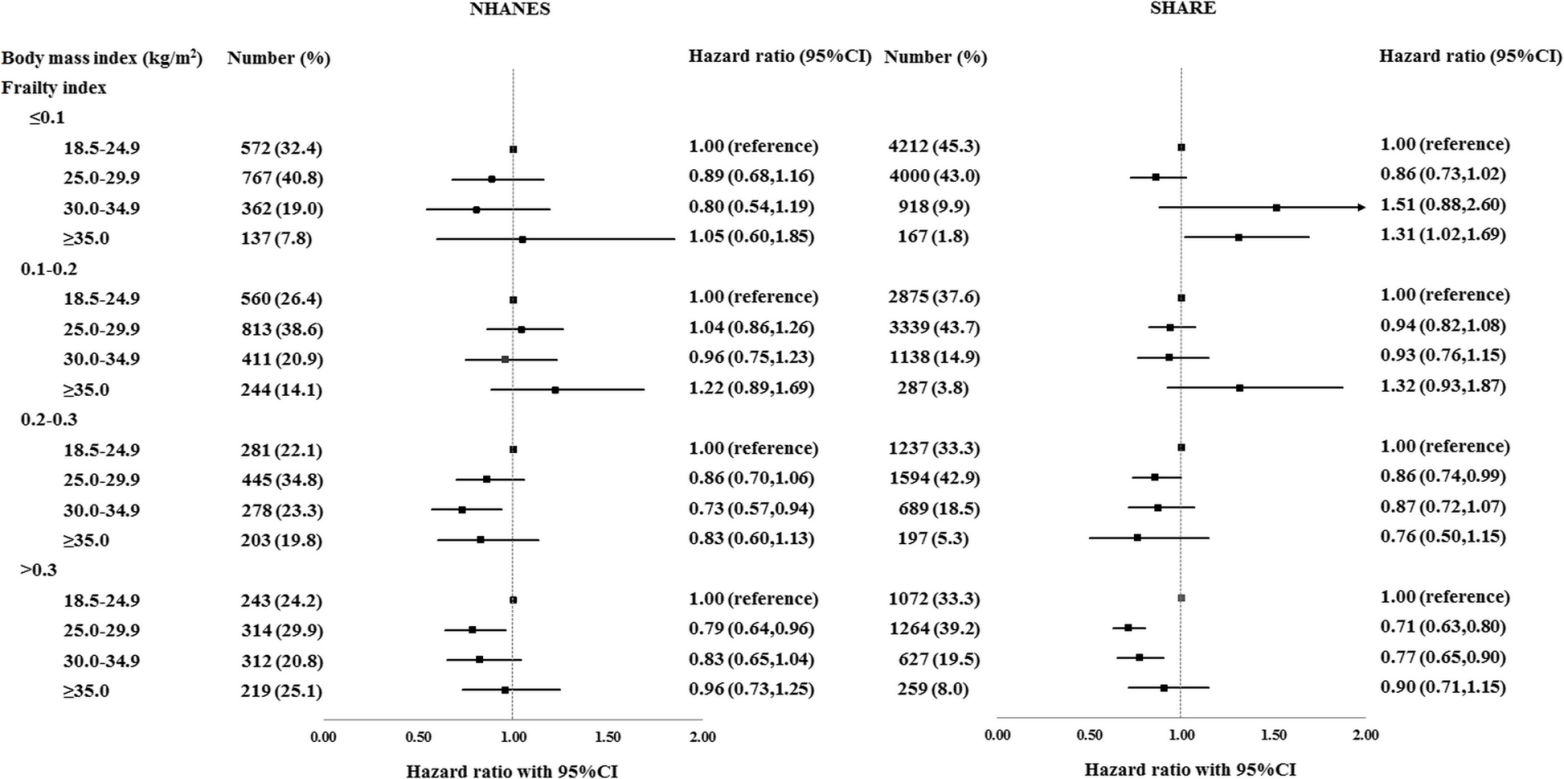
Relationship between body mass index and 10-year mortality risk, stratified by frailty levels. All regression models were adjusted for age, sex, educational level, marital status, employment status, and smoking

We found a significant interaction between frailty and BMI levels in relation to mortality risk (*p* =0.041 in BMI 25.0–29.9 kg/m^2^ (NHANES), and *p* =0.016 in BMI 25.0–29.9 kg/m^2^ and *p* =0.007 in BMI 30.0–34.9 kg/m^2^ (SHARE)) (Additional file 1: Table S3). In participants with FI scores ≤0.1 (SHARE), those with BMI ≥35.0 kg/m^2^ were at statistically significantly higher risk for mortality compared to those with normal BMI. In participants with FI scores 0.2–0.3, those with BMI 25.0–29.9 kg/m^2^ (SHARE) and 30.0–34.9 kg/m^2^ (NHANES) were at statistically significantly lower risk for mortality compared to those with normal BMI, after controlling for the potential covariates. In participants with FI scores >0.3, those having BMI 25.0–29.9 kg/m^2^ in NHANES and those having BMI 25.0–34.9 kg/m^2^ in SHARE were also at a statistically significantly lower risk for mortality (Fig. 2).

### Objective 3: effect of *dual-energy X-ray absorptiometry* (DXA) measured percent body fat as a mediator in the relationships of BMI with frailty and mortality

**Fig. 3:**
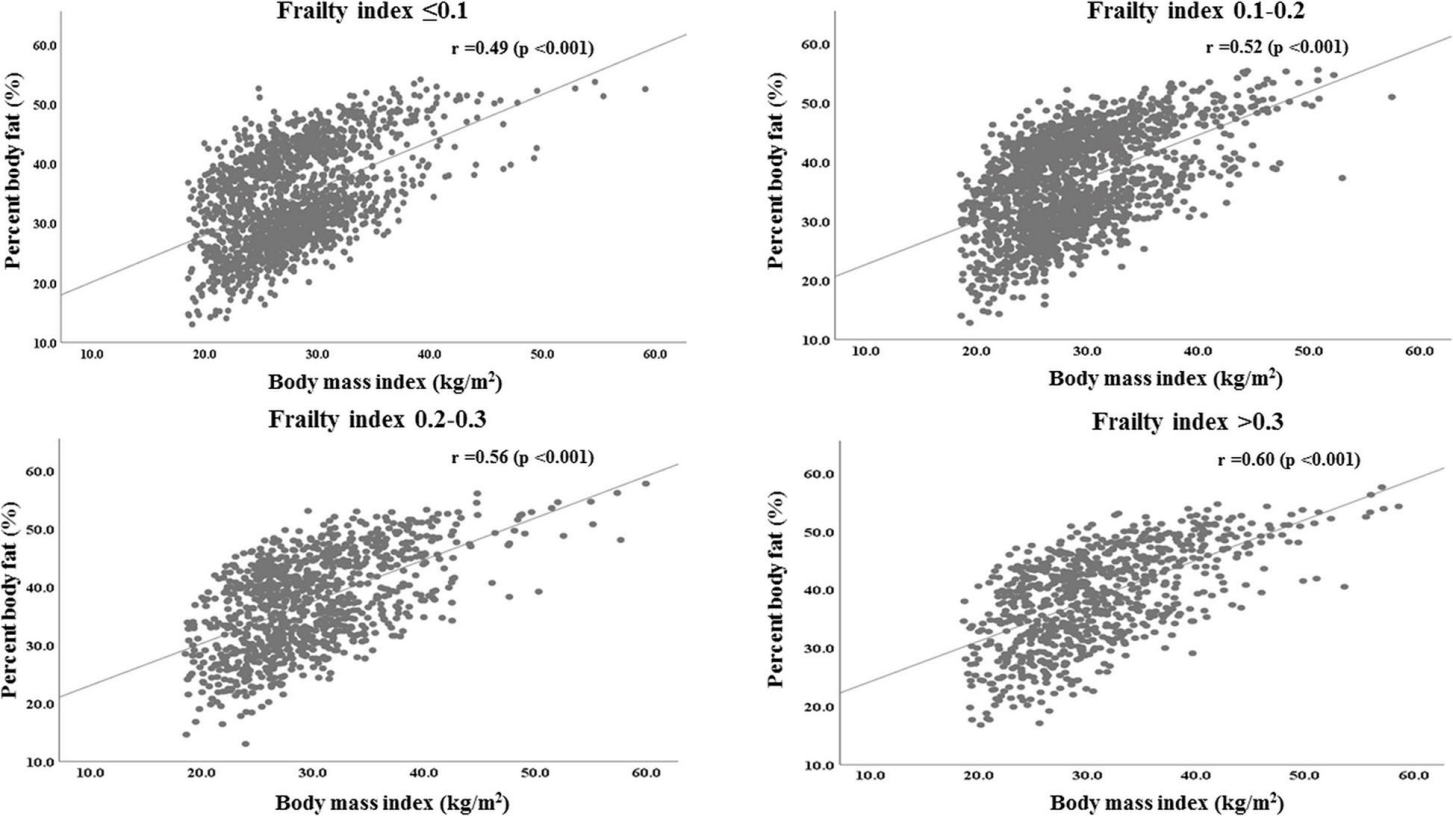
Correlation between BMI and percent body fat, stratified by frailty levels. Data from the 2001–2006 cohorts of NHANES

**Fig. 4:**
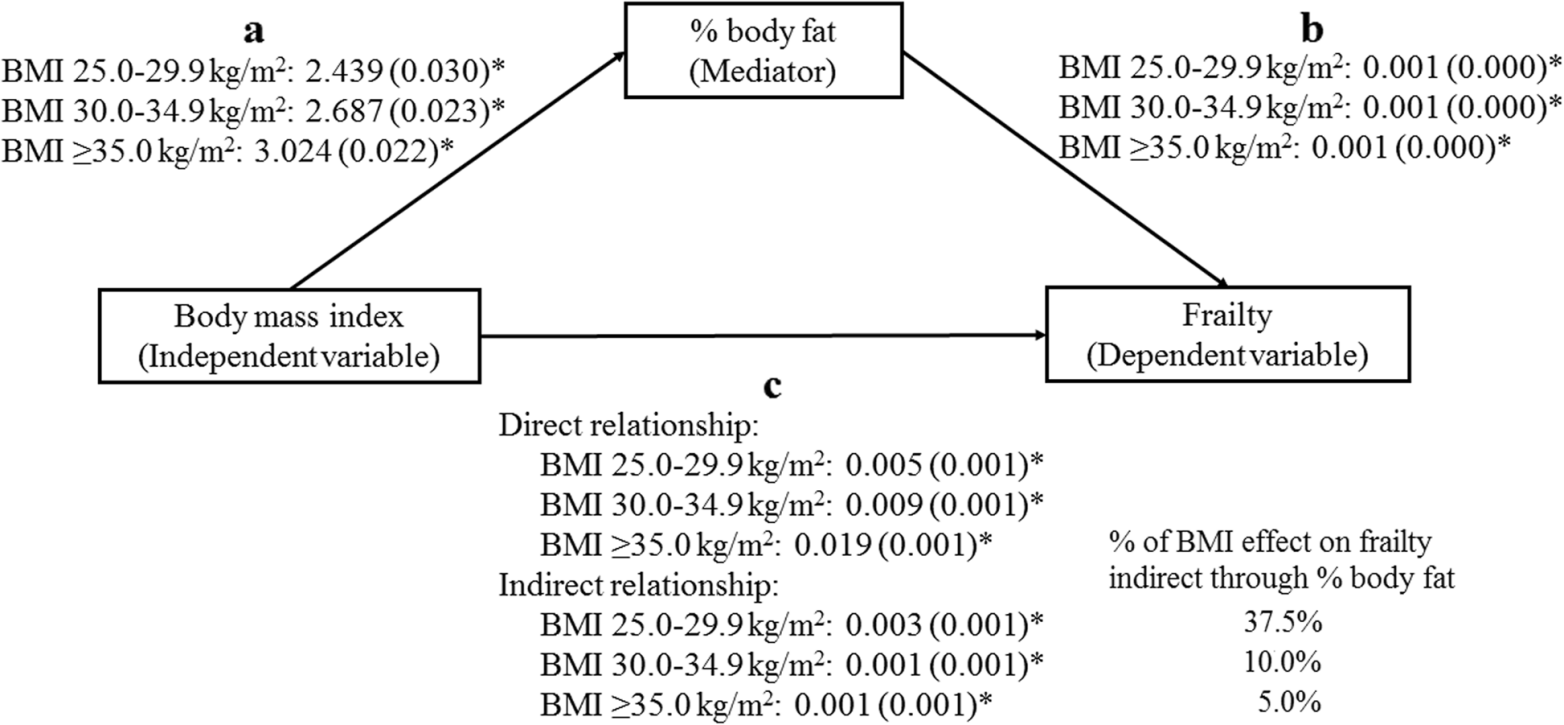
Mediating effect of percent body fat on the relationship between body mass index and frailty, using single-mediation regression model by Andrew F. Hayes. * *p* <0.001. Participant with body mass index 18.5–24.9 kg/m^2^ as a reference group. The numbers were presented in beta-coefficients (standard error). All regression models were adjusted for age, sex, educational level, marital status, employment status, and smoking. Data from the 2001–2006 cohorts of NHANES

In the overall NHANES sample, percent body fat was significantly associated with BMI (*r* =0.54, *p* <0.001) (data not shown in Tables and Figures). This correlation was stronger among people with higher levels of frailty (Fig. 3). The positive association between percent body fat and BMI was also found after adjusting for covariates (Additional file 1: Table S4). Furthermore, higher percent body fat was significantly associated with higher frailty when adjusting for covariates (Table 2). After additionally controlling for BMI, percent fat mass was still positively associated with frailty (Table 2). The single-mediator model revealed statistically significant direct and indirect effects of BMI on frailty. The percentages of the total effect from BMI 25.0–29.9, 30.0–34.9, and ≥35.0 kg/m^2^ to frailty that were indirect through percent body fat were 37.5%, 10%, and 5%, respectively (Fig. 4).

**Table 2:** Relationship of body mass index and percent body fat with frailty (*N* =5309)

Model		Frailty		
		Beta-coefficient (95%CI)	SE	*p*-value
1	Percent body fat (%)	0.003 (0.003,0.004)	0.000	<0.001
2	Percent body fat (%)	0.001 (0.000,0.011)	0.000	<0.001
	Body mass index (kg/m^2^)			
	18.5–24.9 (reference)			
	25.0–29.9	0.005 (0.001,0.009)	0.001	<0.001
	30.0–34.9	0.009 (0.005,0.013)	0.001	<0.001
	≥35.0	0.019 (0.015,0.023)	0.001	<0.001

All regression models were adjusted for age, sex, educational level, marital status, employment status, and smoking. Data from the 2001–2006 cohort of NHANES.

**Fig. 5:**
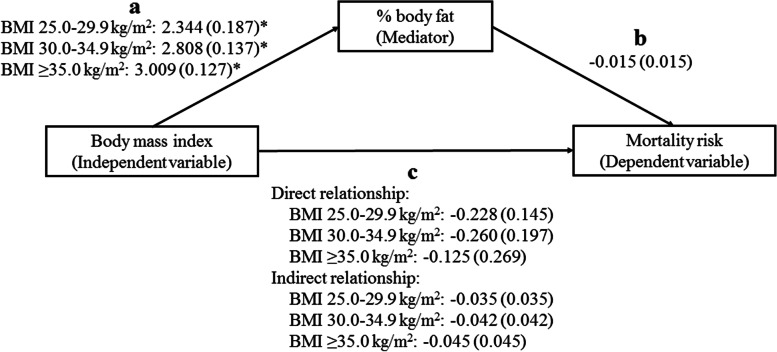
Mediating effect of percent body fat on the relationship between body mass index and mortality in participants with FI 0.2–0.3, using the product of coefficients approaches by Mackinnon. *p <0.001. Participants with body mass index 18.5–24.9 kg/m^2^ as a reference group. Ordinary least squares regression analyses were used to examine the relationship between body mass index and percent body fat. Cox regression analyses were used to examine the relationship of body mass index and percent body fat with mortality risk. The numbers were presented in beta-coefficients (standard error). All regression models were adjusted for age, sex, educational level, marital status, employment status, and smoking. Data from the 2001–2006 cohorts of NHANES

Higher percent body fat was significantly associated with lower mortality risk, in participants with FI 0.2–0.3, when adjusting for covariates. However, after controlling for BMI, percent fat mass was not associated with mortality risk at any level of frailty (Table 3). We did not find a mediated effect of percent body fat on the relationship between higher BMI levels and mortality risk (Fig. 5 and Additional file 1: Table S4).

**Table 3:** Relationship between percent body fat and mortality, and between body mass index and mortality after controlling for percent body fat, using cox regression analysis and stratified by frailty levels (*N* =5309)

Frailty index	Model		Mortality risk			
			Beta-coefficient	SE	Hazard ratio (95%CI)	*p*-value
≤0.1	1	Percent body fat (%)	− 0.012	0.015	0.99 (0.96,1.02)	0.437
	2	Percent body fat (%)	0.008	0.022	1.01 (0.97,1.05)	0.708
		Body mass index (kg/m^2^)				
		18.5–24.9 (reference)				
		25.0–29.9	− 0.272	0.205	0.76 (0.51,1.14)	0.185
		30.0–34.9	− 0.532	0.306	0.59 (0.32,1.07)	0.083
		≥35.0	− 0.223	0.454	0.80 (0.33,1.95)	0.623
0.1–0.2	3	Percent body fat (%)	− 0.007	0.010	0.99 (0.97,1.01)	0.521
	4	Percent body fat (%)	− 0.014	0.013	0.99 (0.96,1.01)	0.308
		Body mass index (kg/m^2^)				
		18.5–24.9 (reference)				
		25.0–29.9	− 0.004	0.139	1.00 (0.76,1.31)	0.977
		30.0–34.9	0.028	0.197	1.03 (0.70,1.51)	0.887
		≥35.0	0.328	0.257	1.39 (0.84,2.30)	0.201
0.2–0.3	5	Percent body fat (%)	− 0.024	0.010	0.98 (0.96,0.99)	0.019
	6	Percent body fat (%)	− 0.015	0.015	0.99 (0.96,1.01)	0.298
		Body mass index (kg/m^2^)				
		18.5–24.9 (reference)				
		25.0–29.9	− 0.228	0.145	0.80 (0.60,1.06)	0.116
		30.0–34.9	− 0.260	0.197	0.77 (0.52,1.14)	0.188
		≥35.0	− 0.125	0.269	0.88 (0.52,1.50)	0.642
>0.3	7	Percent body fat (%)	− 0.011	0.009	0.99 (0.97,1.01)	0.221
	8	Percent body fat (%)	− 0.010	0.013	0.99 (0.97,1.02)	0.459
		Body mass index (kg/m^2^)				
		18.5–24.9 (reference)				
		25.0–29.9	− 0.183	0.140	0.83 (0.63,1.10)	0.189
		30.0–34.9	0.007	0.180	1.01 (0.72,1.44)	0.967
		≥35.0	− 0.095	0.226	0.91 (0.58,1.42)	0.673

All regression models were adjusted for age, sex, educational level, marital status, employment status, and smoking. Data from the 2001–2006 cohort of NHANES

## Discussion

This observational study included data from 6062 US and 23,875 European middle-aged and older adults. We aimed to evaluate the relationships of high BMI with frailty as well as mortality across degrees of frailty and to explore the effect of body fat on these relationships. In both cohorts, overweight (BMI 25.0–29.9 kg/m^2^), obesity grade 1 (30.0–34.9 kg/m^2^), and obesity grade 2 or 3 (≥35.0 kg/m^2^) were associated with higher frailty cross-sectionally and at 10-year follow-up, whereas overweight status was associated with lower mortality risk in moderately/severely frail participants compared to normal BMI. Obesity grade 1 was associated with a lower risk of mortality for people with at least a mild level of frailty and obesity grade 2 or 3 was associated with a higher risk of mortality in non-frailty people but results were not consistent among the two datasets examined. Concerning body fat measured by DXA, higher percent body fat was associated with higher frailty but was not with mortality across frailty levels after controlling for BMI. Percent body fat also had mediating effects on the relationship between BMI and frailty but not on the relationship between BMI and mortality. More than one third of the relationship between BMI and frailty was explained by body fat in overweight people but a smaller part of this relationship was explained by body fat in obese people.

This study revealed that overweight and obesity status were associated with higher frailty, compared to those with normal BMI. A previous meta-analysis [42] also showed that older people having BMI ≥35.0 kg/m^2^ had a higher probability of frailty. The main mechanisms potentially linked between adiposity and adverse outcomes are metabolic changes and mechanical load. Increase in adipocytes, especially intraabdominal fat, causes metabolic syndrome including insulin resistance, type 2 diabetes, hypertension, and dyslipidemia. Moreover, fat cells are related with inflammation, oxidative stress, and immune dysregulation [43, 44] which are the pathological factors of cardiovascular diseases, cognitive impairment, stroke, and cancers [45, 46]. Additionally, increase in body weight causes musculoskeletal degeneration, for example, osteoarthritis of knees, low back pain, and plantar fasciitis [47, 48]. Overweight and obese people also have an increased risk of depression [49]. Not only can shrinking or underweight people be frail, but the accumulation of these obesity-related diseases and conditions also increases health deficits that increases risk of frailty in the future.

The present study showed that being overweight was associated with lower mortality risk in moderately/severely frail participants, compared to people with normal BMI. The inverse association between overweight status and all-cause mortality replicates those found in previous studies, especially in frail people [50, 51]. Body weight is composed of muscle mass, fat mass, bone mineral mass, and body water. Increased BMI can be caused by an increase in any of these body compositions. People with high skeletal muscle mass and strength have a better quality of life and lower mortality rate [52, 53]; on the other hand, sarcopenia increases infection rates, length of hospital stay, immobilization, and mortality [54, 55]. High bone mineral mass is associated with decreased risk of fracture, fracture-related complications, and mortality [18]. However, weight loading may cause and worsen osteoarthritis. In older people, unintentional weight loss produces malnutrition and immune dysfunction that influences adverse outcomes including infection, pressure ulcer, morbidity, and mortality [56, 57]. In patients with comorbidities such as cancer, chronic kidney diseases, chronic obstructive pulmonary disease, and chronic heart failure, low lean mass is a predictor of mortality [58–61]. Mild-grade adiposity can be a protective factor of mortality in older people [62]. Overweight people may also change their lifestyle or more often access medical service when they are frail that influences better control of health deficits and earlier detection of abnormalities. Similar to previous studies [50, 51, 63], our study showed that obesity grades 2 and 3 were associated with increased all-cause mortality in non-frail European participants compared to normal BMI. Obesity increases risks of cardiovascular diseases as well as cardiovascular mortality [64]. Furthermore, levels of inflammatory cytokines were increased in obese people [65]. Prolonged exposure to high levels of inflammation leads to metabolic and immune dysregulation that can cause lethal conditions [43, 44] and may overcome protective effects on mortality in severely obese people. By these reasons, being overweight may be a protective factor of mortality in frail people but severe obesity could increase risk of mortality even if they are fit. Being overweight may be protective of mortality but it may be positively associated with other adverse health outcomes such as pain and quality of life.

Our study found that percent body fat was independently positively associated with frailty and that the relationship between BMI and frailty was mediated by percent body fat. However, we found no association between percent body fat and frailty after controlling for BMI or a mediating effect of percent body fat on the relationship between BMI and mortality across frailty levels. Adipocytes produce a lot of inflammatory cytokines, for example, tumor necrosis factor-α (TNF-α), monocyte chemoattractant protein-1 (MCP-1), interleukin (IL-6), leptin, and resistin [66]. Inflammation is an important factor related to cardiovascular diseases (e.g., atherosclerosis, insulin resistance, and hypercoagulable state) and non-cardiovascular diseases (e.g., cancer, sarcopenia, chronic obstructive pulmonary disease, renal diseases, and depression) [65]. Due to the adverse effects of adiposity, higher percent body fat increases health deficit accumulation. Nonetheless, the relationship between body fat and mortality risk is still inconsistent [67, 68]. Distribution of body fat may be a better predictor of mortality than actual fat mass. Increased visceral fat area and visceral-to-subcutaneous fat area ratio were associated with increased all-cause mortality [69]. In this study, waist circumference was moderately correlated with percent body fat and there was no association between waist circumference and mortality across frailty levels after controlling for BMI (data were not presented).

We analyzed publicly available data from two large population-based cohorts with well-controlled and rigorous protocols. Moreover, the FIs created from NHANES and SHARE are well-established. For both datasets, 10-year all case mortality was examined, and SHARE also allowed us to examine frailty longitudinally. The findings of this study can challenge the view that frailty is only a shrinking syndrome. Patients with a high BMI can also be frail. However, there are some aspects of this study that should be considered when interpreting the results. Firstly, BMI can change over time and may affect clinical outcomes. Secondly, frailty can change over time and health-related behaviors may also change when people become frailer. Thirdly, body fat was only measured in one of the two included datasets, so future studies should examine further whether body fat mediates the relationship between frailty and BMI. Also, future studies of the mechanisms of body composition are warranted to explain further the relationship of body fat with frailty and mortality. The impact of frailty and BMI on CVD and cancer mortality should also be examined. Fourthly, participation bias, common in population studies, may have impacted our results. Lastly, we should note that there were some differences in characteristics between the two datasets such as that SHARE participants had lower education level and income than NHANES participants.

## Conclusions

This study revealed that overweight and obesity status were associated with higher frailty levels both cross-sectionally and longitudinally. Obesity grades 2 and 3 could be associated with higher mortality risk in non-frail people, whereas being overweight was a protective factor of mortality in moderately/severely frail people. Percent body fat is a mediator of the relationship between BMI and frailty.

## Supplementary Information

**Additional file 1: Table S1.** [36-item Frailty Index (NHANES)]. **Table S2.** [57-item Frailty Index (SHARE)]. **Table S3.** [Interactions between frailty and BMI levels in relation to mortality risk]. **Table S4.** [Relationship between body mass index and percent body fat, using ordinary least squares regression analysis, and between body mass index and mortality, using cox regression analysis and stratified by frailty levels (*N* =5309)].

## Data Availability

The National Health and Nutrition Examination Survey (NHANES) data are publicly available at https://www.cdc.gov/nchs/nhanes/index.htm. The Survey of Health, Ageing and Retirement in Europe (SHARE) data are available at https://www.share-project.org

## Abbreviations

BMI: Body mass index
DXA: Dual-energy X-ray absorptiometry
FI: Frailty index
NHANES: National Health and Nutrition Examination Survey
SHARE: Survey of Health, Ageing and Retirement in Europe
