# Supplementary material for: Relationship of body mass index with frailty and all-cause mortality among middle-aged and older adults
Source: BMC Med. 2022 Oct 24;20:404. doi: 10.1186/s12916-022-02596-7 (PMC9594976; doi:10.1186/s12916-022-02596-7)
Supplement: Supplementary file 1 — Additional file 1: Table S1. [36-item Frailty Index (NHANES)]. Table S2. [57-item Frailty Index (SHARE)]. Table S3. [Interactions between frailty and BMI levels in relation to mortality risk]. Table S4. [Relationship between body mass index and percent body fat, using ordinary least squares regression analysis, and between body mass index and mortality, using cox regression analysis and stratified by frailty levels (N =5309)]. [file 12916_2022_2596_MOESM1_ESM.docx]

**Table S1. 36-item Frailty Index (NHANES)**

| **Self-reported Frailty Index items** | |
| --- | --- |
| 1. Angina/angina pectoris | 14. Difficulty lifting or carrying |
| 2. Heart attack | 15. Difficulty walking between rooms on same floor |
| 3. Coronary heart disease | 16. Difficulty standing up from an armless chair |
| 4. Stroke | 17. Difficulty getting in and out of bed |
| 5. Thyroid condition | 18. Difficulty dressing yourself |
| 6. Cancer | 19. Difficulty grasping/holding small objects |
| 7. Arthritis | 20. Difficulty attending social events |
| 8. High blood pressure | 21. Self-reported health |
| 9. Diabetes mellitus | 22. Frequency of healthcare use |
| 10. Weak/failing kidneys | 23. Health compared to 1 year ago |
| 11. Confusion or inability to remember things | 24. Overnight hospital stays |
| 12. Difficulty managing money | 25. Medications |
| 13. Difficulty stooping, crouching, kneeling |  |
| **Laboratory Frailty Index items** | |
| 26. Pulse rate (60-99 bpm) | 32. Red cell distribution width (≤14.6%) |
| 27. Systolic blood pressure (90-140 mmHg) | 33. Lactate dehydrogenase (≤190 U/L) |
| 28. Pulse pressure (30-60 mmHg) | 34. Alkaline phosphatase (≤115 U/L) |
| 29. Platelet count SI (150-450 unit 1000 cells/uL) | 35. Uric acid (M: 240-510, F: 160-430 umol/L) |
| 30. Blood urea nitrogen (3-20 mg/dL) | 36. Total calcium (2.0-2.5 mmol/L) |
| 31. Bicarbonate (≤28 mmol/L) |  |

F = female; M = male

**Table S2. 57-item Frailty Index (SHARE)**

| 1. Self-rated health | 31. Walking across a room |
| --- | --- |
| 1. Hospitalization | 32. Getting up from a chair |
| 1. Heart Attack | 33. Bathing or showering |
| 1. Stroke | 34. Eating |
| 1. High blood cholesterol | 35. Getting in or out of bed |
| 1. Diabetes | 36. Preparing a hot meal |
| 1. Chronic Lung Disease | 37. Using the toilet |
| 1. Long-term illness | 38. Using map to get around |
| 1. Arthritis | 39. Making telephone calls |
| 1. Cancer | 40. Taking medications |
| 1. High blood pressure | 41. Shopping for groceries |
| 1. Stomach or duodenal ulcer | 42. Managing money |
| 1. Parkinson Disease | 43. Vigorous activity |
| 1. Cataracts | 44. Moderate Activity |
| 1. Hip or femoral fracture | 45. Doing work around house or garden |
| 1. Falling down | 46. Limitations with activities |
| 1. Sleep problems | 47. Orientation |
| 1. Dizziness | 48. Suicidality |
| 1. Eyesight | 49. Trouble sleeping |
| 1. Hearing | 50. Depression |
| 1. Pain in any joint | 51. Interest |
| 1. Climbing several flights of stairs | 52. Appetite |
| 1. Stooping/kneeling/crouching | 53. Fatigue |
| 1. Sitting for 2 hours | 54. Pessimism |
| 1. Reaching or extending arms | 55. Concentration |
| 1. Pulling or pushing large objects | 56. Lack of enjoyment |
| 1. Lifting or carrying weight >5kg | 57. Fear of falling down |
| 1. Walking 100 metres |  |
| 1. Picking up small coin from a table |  |
| 1. Dressing |  |

**Table S3. Interactions between frailty and BMI levels in relation to mortality risk**

| Interaction | | Mortality | | | | | |
| --- | --- | --- | --- | --- | --- | --- | --- |
|  |  | NHANES | | | SHARE | | |
|  |  | Beta-coefficient | SE | *p*-value | Beta-coefficient | SE | *p*-value |
| Frailty Index | Body mass index (kg/m^2^) |  |  |  |  |  |  |
|  | 18.5-24.9 (reference) |  |  |  |  |  |  |
|  | 25.0-29.9 | -3.659 | 1.361 | 0.041 | -0.079 | 0.033 | 0.016 |
|  | 30.0-34.9 | 1.789 | 2.893 | 0.536 | -0.128 | 0.047 | 0.007 |
|  | ≥35.0 | 4.524 | 3.488 | 0.195 | -0.126 | 0.086 | 0.142 |

Regression model included age, sex, educational level, marital status, employment status, smoking, frailty index, and body mass index.

**Table S4. Relationship between body mass index and percent body fat, using ordinary least squares regression analysis,** **and between body mass index and mortality, using cox regression analysis and stratified by frailty levels (N =5309)**

| Frailty index |  | Percent body fat (%) | | | Mortality risk | | | |
| --- | --- | --- | --- | --- | --- | --- | --- | --- |
|  |  | Beta-coefficient (95%CI) | SE | *p*-value | Beta-coefficient | SE | Hazard ratio  (95%CI) | *p*-value |
| ≤0.1 | Body mass index (kg/m^2^) |  |  |  |  |  |  |  |
|  | 18.5-24.9 (reference) |  |  |  |  |  |  |  |
|  | 25.0-29.9 | 2.513 (2.293,2.733) | 0.112 | <0.001 | -0.120 | 0.138 | 0.89 (0.68,1.16) | 0.385 |
|  | 30.0-34.9 | 2.714 (2.532,2.896) | 0.093 | <0.001 | -0.219 | 0.201 | 0.80 (0.54,1.19) | 0.278 |
|  | ≥35.0 | 3.103 (2.898,3.309) | 0.105 | <0.001 | 0.050 | 0.290 | 1.05 (0.60,1.85) | 0.863 |
| 0.1-0.2 | Body mass index (kg/m^2^) |  |  |  |  |  |  |  |
|  | 18.5-24.9 (reference) |  |  |  |  |  |  |  |
|  | 25.0-29.9 | 2.462 (2.218,2.705) | 0.124 | <0.001 | 0.043 | 0.097 | 1.04 (0.86,1.26) | 0.658 |
|  | 30.0-34.9 | 2.677 (2.480,2.874) | 0.100 | <0.001 | -0.041 | 0.128 | 0.96 (0.75,1.23) | 0.746 |
|  | ≥35.0 | 2.977 (2.789,3.164) | 0.096 | <0.001 | 0.201 | 0.165 | 1.22 (0.89,1.69) | 0.223 |
| 0.2-0.3 | Body mass index (kg/m^2^) |  |  |  |  |  |  |  |
|  | 18.5-24.9 (reference) |  |  |  |  |  |  |  |
|  | 25.0-29.9 | 2.344 (1.977,2.711) | 0.187 | <0.001 | -0.151 | 0.106 | 0.86 (0.70,1.06) | 0.156 |
|  | 30.0-34.9 | 2.808 (2.540,3.076) | 0.137 | <0.001 | -0.310 | 0.126 | 0.73 (0.57,0.94) | 0.014 |
|  | ≥35.0 | 3.009 (2.759,3.259) | 0.127 | <0.001 | -0.190 | 0.160 | 0.83 (0.60,1.13) | 0.234 |
| >0.3 | Body mass index (kg/m^2^) |  |  |  |  |  |  |  |
|  | 18.5-24.9 (reference) |  |  |  |  |  |  |  |
|  | 25.0-29.9 | 2.379 (1.925,2.832) | 0.231 | <0.001 | -0.242 | 0.103 | 0.79 (0.64,0.96) | 0.019 |
|  | 30.0-34.9 | 2.588 (2.248,2.927) | 0.173 | <0.001 | -0.192 | 0.119 | 0.83 (0.65,1.04) | 0.107 |
|  | ≥35.0 | 2.990 (2.734,3.246) | 0.130 | <0.001 | -0.045 | 0.135 | 0.96 (0.73,1.25) | 0.741 |

All regression models were adjusted for age, sex, educational level, marital status, employment status and smoking. Data from 2001-2006 cohort of NHANES.
